# Retraction: Expression of microRNAs and their target genes in melanomas originating from gynecologic sites

**DOI:** 10.1371/journal.pone.0339074

**Published:** 2025-12-18

**Authors:** The PLOS One Editors

The *PLOS One* Editors retract this article [1] because it was identified as one of a series of submissions for which we have concerns about compromised peer review.

The Editors have no evidence of any author involvement in the peer review concerns.

WEC did not agree with the retraction. MJDV, CDA, LPSK, CR, ZB, MM, PF, LY, FJB, KR, AM, CS, CC, and AAG either did not respond directly or could not be reached.
